# Correction: Psychological distress and burnout among healthcare worker during COVID-19 pandemic in India—A cross-sectional study

**DOI:** 10.1371/journal.pone.0314925

**Published:** 2024-11-27

**Authors:** Geetha R. Menon, Jeetendra Yadav, Sumit Aggarwal, Ravinder Singh, Simran Kaur, Tapas Chakma, Murugesan Periyasamy, Chitra Venkateswaran, Prashant Kumar Singh, Rakesh Balachandar, Ragini Kulkarni, Ashoo Grover, Bijaya Kumar Mishra, Maribon Viray, Kangjam Rekha Devi, K. H. Jitenkumar Singh, K. B. Saha, P. V. Barde, Beena Thomas, Chandra Suresh, Dhanalakshmi A., Basilea Watson, Pradeep Selvaraj, Gladston Xavier, Denny John, Jaideep Menon, Sairu Philip, Geethu Mathew, Alice David, Raman Swathy Vaman, Abey Sushan, Shalini Singh, Kiran Jakhar, Asha Ketharam, Ranjan Prusty, Jugal Kishore, U. Venkatesh, Subrata Kumar, Srikanta Kanungo, Krushna Sahoo, Swagatika Swain, Anniesha Lyngdoh, Jochanan Diengdoh, Phibawan Syiemlieh, AbuHasan Sarkar, Gajanan Velhal, Swapnil Kharnare, Deepika Nandanwar, M. Vishnu Vardhana Rao, Samiran Panda

There are errors in the author affiliations. The correct affiliations are as follows:

Geetha R. Menon^1^ , Jeetendra Yadav^1^ , Sumit Aggarwal^2^, Ravinder Singh^2^, Simran Kaur^1^ , Tapas Chakma^3^, Murugesan Periyasamy^4^, Chitra Venkateswaran^5^, Prashant Kumar Singh^6^, Rakesh Balachandar^7^, Ragini Kulkarni^8^, Ashoo Grover^9^, Bijaya Kumar Mishra^10^, Maribon Viray^11^, Kangjam Rekha Devi^12^, K. H. Jitenkumar Singh^1^, K. B. Saha^3^, P. V. Barde^3^, Beena Thomas^4^, Chandra Suresh^4^, Dhanalakshmi A.^4^, Basilea Watson^4^, Pradeep Selvaraj^13^, Gladston Xavier^14^, Denny John^15^, Jaideep Menon^15^, Sairu Philip^16^, Geethu Mathew^5^, Alice David^5^, Raman Swathy Vaman^17^, Abey Sushan^18^, Shalini Singh^6^, Kiran Jakhar^19^, Asha Ketharam^7^, Ranjan Prusty^8^, Jugal Kishore^20^, U. Venkatesh^20^, Subrata Kumar^10^, Srikanta Kanungo^10^, Krushna Sahoo^10^, Swagatika Swain^10^, Anniesha Lyngdoh^11^, Jochanan Diengdoh^11^, Phibawan Syiemlieh^11^, AbuHasan Sarkar^12^, Gajanan Velhal^21^, Swapnil Kharnare^22^, Deepika Nandanwar^21^, M. Vishnu Vardhana Rao^1^, Samiran Panda^2^

**1** Indian Council of Medical Research- National Institute of Medical Statistics, New Delhi, India, **2** Indian Council of Medical Research, New Delhi, India, **3** Indian Council of Medical Research- National Institute of Research in Tribal Health, Jabalpur, Madhya Pradesh, India, **4** Indian Council of Medical Research- National Institute of Research in Tuberculosis, Chennai, Tamil Nadu, India, **5** Believers Church Medical College Hospital, Tiruvalla, Kerala, India, **6** ICMR-National Institute of Cancer Prevention and Research, Gautam Buddh Nagar, Uttar Pradesh, India, **7** ICMR-National Institute of Occupational Health, Ahmedabad, Gujarat, India, **8** ICMR- National Institute of Research in Reproductive Health, Mumbai, Maharashtra, India, **9** ICMRNational Institute of Pathology, New Delhi, India, **10** Regional Medical Research Centre, Bhubaneswar, Odisha, India, **11** Martin Luther Christian University, Shillong, Meghalaya, India, **12** ICMR -Regional Medical Research Centre, Dibrugarh, Assam, India, **13** Directorate of public health and Preventive Medicine, Chennai, Tamil Nadu, India, **14** Loyola College, Chennai, Tamil Nadu, India, **15** Amrita Institute of Medical Sciences & Research Centre, Kochi, Kerala, India, **16** Professor, Department of Community Medicine, Govt T D Medical College, Alappuzha, Kerala, **17** District Program Manager, National Health Mission, Kasargode, Kerala, **18** District Program Manager, National Health Mission, Pathanamthitta, Kerala, **19** Government Institute of Medical Sciences, Greater Noida, Uttar Pradesh, India, **20** Vardhman Mahavir Medical College and Safdarjung Hospital, New Delhi, India, **21** Seth GS Medical College and KEM Hospital, Mumbai, Maharashtra, India, **22** Hinduja Hospital, Mumbai, Maharashtra, India.
