# Supplementary material for: Psychological distress and burnout among healthcare worker during COVID-19 pandemic in India—A cross-sectional study
Source: PLoS One. 2022 Mar 10;17(3):e0264956. doi: 10.1371/journal.pone.0264956 (PMC8912126; doi:10.1371/journal.pone.0264956)
Supplement: S1 File — (DOCX) [file pone.0264956.s001.docx]

S1 (Questionnaire)

1/14

**ICMR STUDY ON MENTAL HEALTH AND
SOCIAL STIGMA AMONG HEALTHCARE
PERSONNEL INVOLVED IN THE
MANAGEMENT OF COVID-19 PATIENTS
IN INDIA**

Part I

Dear Participant,

You are invited to be part of a research study “Mental health and social stigma

among healthcare personnel involved in the management of COVID-19 patients in
India” conducted by the Indian Council of Medical Research and its coordinating
institutes. Please find below the relevant information regarding the study for you to
make a voluntary decision regarding your participation in the study. You are
requested to ask any questions regarding the study for the necessary clarifications.

Information about the Research study:

COVID-19 is the worst epidemic in the 21st century. The health care personnel

involved in the management and treatment of COVID-19 suspected/confirmed cases
have a crucial role in the revival of the current situation. While it is realized that
there are high rates of transmission among health care workers and the need for
prevention strategies in this regard little is known about the mental health issues
that health care providers face. This may include emotional exhaustion, physical
distress leading to anxiety, problems of balancing family and work life, stigma at
various levels among others. This could have an influence on the health care
performance of the providers which is of paramount importance in the management
of COVID 19. The findings will help understand these issues which are crucial and
help develop mental health intervention strategies that need to be provided for health
care providers to equip themselves in optimum care for patients as well as balance
the challenges around work life and family.

The purpose of the study is to assess mental health and social stigma among healthcare workers involved in the management of COVID-19 patients.

Study Procedures:

If you are willing to take part in this research study, you will have to answer questions related to your demography (personal details), work, working conditions and your experience at work. The entire procedure would take about 45 minutes

Benefits of the study

• Data on factors promoting positive mental health and negative mental health of your workplace will be available.

**Participant
Information
Sheet**

• Data shall help stakeholders (government, institutions, organizations) to draft policies on protecting the mental health of healthcare workers (if necessary).

Risks involved

The only risk that you may be concerned about is the concerns if the information
given would affect your relationship with the health care officials. Care would be
taken that this information is kept confidential with no identifiers that would affect
your job profile. Furthermore, the interviews would be on the telephone and care

would be taken to see that all privacy is strictly maintained. You may also find some
of the questions sensitive in nature. We understand that this may be difficult and you
may feel free to take your time and skip any questions you do not want to respond to.

Taking part in this study is voluntary. You may withdraw from the study without having to provide a reason.

Privacy and confidentiality

You will be assigned a unique identification number (ID) to be used on study forms,
in the study database None of the study forms or study databases will contain your/
name or other information that could be used to identify you. The document linking
your IDs to your name and medical record numbers will be kept in a locked office and
will not be accessible to personnel not associated with the study. Your identity will
remain confidential, except as required by law. Data derived from the study will be

coded, with only the Principal Investigator or his/her designated research colleagues having access to the code. Any scientific presentation or publication will not reveal your identity individually. You may choose to deny providing any information which you may feel uncomfortable sharing during your participation in the study.

Contact persons

During the study period, you need any counseling or consultation to a specialist, you will be referred to an appropriate specialist.

2/14

If you have any questions regarding this study, you can contact Dr. Tapas Chakma, Scientist G 0761-2672557

If you are willing to participate in this study, please sign the Informed Consent Form, consenting to participate.

I have read the information sheet of the study titled ‘Mental health and social stigma
among healthcare personnel involved in the management of COVID-19 patients in India
and have discussed with the study investigators about the purpose of the study, the
procedures involved, the anticipated risks and benefits involved, subject safety
procedures and protection of rights of study subjects. I have been given the opportunity
to ask questions, which have been answered to my satisfaction. I understand that my
participation in this study is voluntary and that I may refuse to participate. I also
understand that if, for any reason, I wish to discontinue participation in this study at any

**Consent**

**Form**

time, I will be free to do so.I understand that as a participant in this study, my identity,
medical records, and data relating to this research study will be kept confidential, except
as required by law. If I have any questions concerning my rights as a research subject in
this study, I may contact any of the study investigators at any time point. As I am fully

informed of the study, its risks and benefits, I hereby consent to the procedures set forth.

Declaration

I have been read out the consent form and I understand all the information given. 'I have been made aware of the research study in detail, I understand and I give my consent to participate in the study" .

1. Consent given *

*Mark only one oval.*

No

Yes *Skip to question 2*

**Implementation agency details**

3/14

2. Implementing agency *

*Mark only one oval.*

NIRRH Mumbai

NIOH Ahmedabad
NIP Delhi

NICPR Noida

NIRTH Jabalpur

RMRC Bhubaneswar NIRT Chennai

BMCH Tiruvalla

RMRC Diburugarh

Martin Luther Christian Univ

3. State

*Mark only one oval.*

Maharashtra

Gujarat

Uttar Pradesh

Madhya Pradesh
Delhi

Odisha
Assam

Meghalaya
Tamil Nadu
Kerala

4/14

4. District/city *

*Mark only one oval.*

Mumbai

Ahmedabad

South East Delhi
Noida

Jabalpur

Bhubaneswar
Cuttack

Chennai

Ernakulam

Pattanamthitta
Kamrup

East Khasi Hills

**Health facility details**

5. Name of the facility (optional)

6. Location of the facility *

7. Type of Facility *

*Mark only one oval.*

Public

Private

Any other

5/14

8. Average number of COVID patients screened per day

9. Average Number COVID patients under care per day

**Socioeconomic Details**

10. Name(Optional)

11. Age (In Complete Years) *

12. Sex *

*Mark only one oval.*

Male

Female

Transgender

Prefer not to respond

13. Marital status *

*Mark only one oval.*

Unmarried

Married

Separated/Divorcee
Widow

6/14

14. Education *

*Mark only one oval.*

Upto Middle school High School

Higher secondary
Diploma in nursing
Graduate

Post Graduate and above

15. Location

*Mark only one oval.*

Urban

Rural

Semi urban/slum

16. Family members (Number):

17. Residing with family *

*Mark only one oval.*

Yes

No

7/14

18. Monthly income of the respondent (optional)

*Mark only one oval.*

<INR 5000

5000-10000 INR
10000-20000 INR
20000-50000 INR
>50000 INR

19. Institutional workers *

*Mark only one oval.*

Yes

No *Skip to question 22*

If institutional worker

20. Designation *

*Mark only one oval.*

Doctor

Nurse

Auxiliary nurse / paramedical staff
Laboratory staff - processing
Laboratory staff- sample collection
Supporting staff- Ambulance driver
Supporting staff - Ambulance workers
Supporting staff-ward boys

X Ray technician

House-keeping- sanitation workers/Guards House keeping- guards

8/14

21. Employment Status *

*Mark only one oval.*

Temporary/adhoc Permanent

*Skip to question 23*

If Non-institutional

22. Designation

*Mark only one oval.*

ASHA

Community health worker
Urban Health Worker

Nature of work

23. Average number of hours of working per day *

24. How many COVID-19 patients/suspects do you see/screen/ see/screen/test/transport/ take

care per day? *

25. No. of working days in a typical week *

9/14

26. Does your work involve one or more of these activities related to COVID patients? *

*Mark only one oval per row.*

YES NO

In quarantine
In Isolation

With symptoms needing care In Intensive Care

In end of life care
In bereavement
Contact Tracing
Community care
Screening

Sample testing

Transport of patients

27. Have you been administered/ self-administered Hydroxychloroquine as a prophylaxis *

*Mark only one oval.*

Yes

No *Skip to question 31*

**If yes to Question 19**

28. a. Dose

10/14

29. b. Duration

30. c. Completion status

*Mark only one oval.*

Yes

No

NA

Part II

The General Health Questionnaire (GHQ) is a screening tool for mass screening of
**patients with psychological morbidity including depression/anxiety Since its**

**General**

**Health**

**Questionnaire (GHQ-5)**

31. Questions *

development by Goldberg in the 1970s (Goldeberg, 1978)) it has been extensively used in different settings and different cultures. The original scale was modified as GHQ-5 and validated. The GHQ-5 has exhibited 86% sensitivity and 89%

specificity in screening individuals with psychiatric illness (Shamsunder et al 1986).

*Mark only one oval per row.*

YES NO

Have you recently lost much sleep over
worry?

Have you recently felt constantly under
strain?

Have you recently been able to enjoy normal day-to-day activities?

Have you recently been feeling reasonably happy all things considered?

Have you recently been feeling unhappy and depressed?

Part III

**Burnout Questionnaire**

11/14

9/7/2020 ICMR-STUDY ON MENTAL HEALTH AND SOCIAL STIGMA AMONG HEALTHCARE PERSONNEL INVOLVED IN THE MANAGEM…

32. Section A: Burnout (Emotional exhaustion) *

*Mark only one oval per row.*

NEVER SOMETIMES ALWAYS

Emotional exhaustion

Demotivated to attend work.

The thought of going to work puts me down. This work disinterests me.

I work here solely for money and the job doesn’t motivate / encourage me

I am worried about attending work

I keep thinking about work related issues even during non-working hours

I feel sleepless, loss of appetite due to continuous thoughts about work

I don’t feel lively while being at work I feel frustrated by my work
I feel satisfied with my work

Thoughts of work prevents me from enjoying happiness with family & friends.

12/14

33. Section B: Depersonalization *

*Mark only one oval per row.*

NEVER SOMETIMES ALWAYS

I express anger at workplace due to work related stress / excess of work

This job is making me uncaring / indifferent / to my fellow colleagues

I’ve become insensitive to people around me, since I’ve been working

I almost lose my patience at the end of my
workday

Feel guilty / responsible for some of the problems of my colleague /client.

34. Section C: Personal accomplishment *

*Mark only one oval per row.*

Never Sometimes Always

I accomplish many worthwhile things in this
job. 1

I satisfactorily complete my work for the day

I am satisfied with my efforts of keeping the work atmosphere calm and relaxed

35. Name of the interviewer *

13/14
