# Supplementary figures and images for: Psychological distress and burnout among healthcare worker during COVID-19 pandemic in India—A cross-sectional study
Source: PLoS One. 2022 Mar 10;17(3):e0264956. doi: 10.1371/journal.pone.0264956 (PMC8912126; doi:10.1371/journal.pone.0264956)

S2. Functional diagram to explain the flow of work

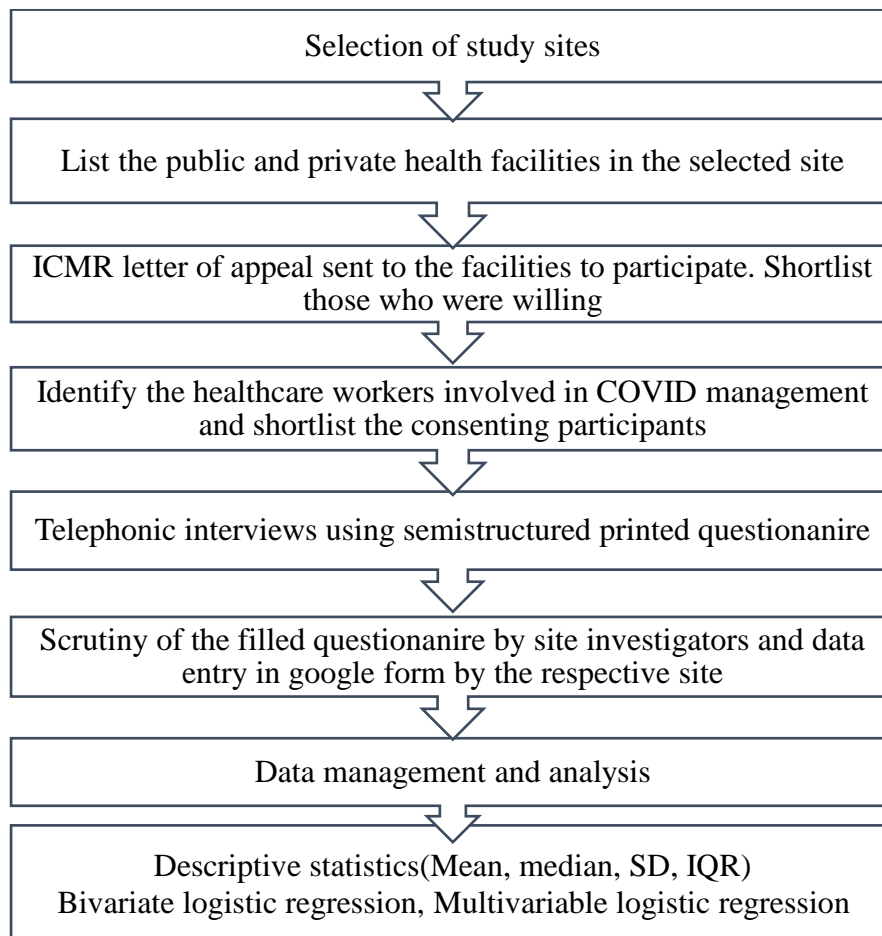

Supplement: S2 File — (PDF) [file pone.0264956.s002.pdf]
