# Supplementary material for: Psychological distress and burnout among healthcare worker during COVID-19 pandemic in India—A cross-sectional study
Source: PLoS One. 2022 Mar 10;17(3):e0264956. doi: 10.1371/journal.pone.0264956 (PMC8912126; doi:10.1371/journal.pone.0264956)
Supplement: S1 Table — (DOCX) [file pone.0264956.s003.docx]

**S2 Table**

**Sociodemographic, job-related characteristics and the burnout subscale scores**

|  | **Emotional exhaustion** | | |  | **Depersonalization** | |  | **Personal accomplishment** | |  |
| --- | --- | --- | --- | --- | --- | --- | --- | --- | --- | --- |
|  | **Mean± S. D** | **Med (IQR)** | | **p-value** | **Mean± S. D** | **Med (IQR)** | **p-value** | **Mean± S. D** | **Med (IQR)** | **p-value** |
| **Age Group** |  |  | |  |  |  |  |  |  |  |
| <=34 years | 15.65 ± 3.80 | 15(5) | | 0.14 | 6.93 ± 1.80 | 7(3) | 0.001 | 8.19 ± 1.01 | 9(2) | 0.001 |
| 35-44 years | 15.13 ± 3.39 | 14(5) | |  | 6.51 ± 1.64 | 6(2.25) |  | 8.22 ± 1.08 | 9(2) |  |
| >=45 years | 14.92 ± 3.48 | 14(5) | |  | 6.07 ± 1.32 | 6(2) |  | 8.48 ± 1.03 | 9(1) |  |
| **Gender** | | | | | | | | |  |  |
| Male | 15.37 ± 3.67 | 15(5) | | 0.53 | 6.70 ± 1.67 | 6(3) | 0.91 | 8.22 ± 1.06 | 9(2) | 0.67 |
| Female | 15.47 ± 3.67 | 15(5) | |  | 6.71 ± 1.77 | 6(3) |  | 8.25 ± 1.01 | 9(2) |  |
| **Marital status** |  | | |  |  | | |  |  |  |
| Married | 15.33 ± 3.60 | 15(5) | | 0.45 | 6.53 ± 1.66 | 6(2) | 0.00 | 8.32 ± 1 | 9(1) | 0.001 |
| Single | 15.56 ± 3.77 | 15(5) | |  | 6.98 ± 1.79 | 7(3) |  | 8.11 ± 1.08 | 9(2) |  |
| **Occupation** |  |  | |  |  |  |  |  |  |  |
| Doctor | 16.36 ± 3.87 | 16(6) | | 0.001 | 7.15 ± 1.85 | 7(2.50) | 0.001 | 7.88 ± 1.22 | 8(2) | 0.001 |
| Auxiliary nurse/paramedical staff | 15.16 ± 3.39 | 15(4) | |  | 6.27 ± 1.53 | 6(2) |  | 8.46 ± 0.86 | 9(1) |  |
| Nurse | 15.82 ± 3.79 | 15(5) | |  | 7.06 ± 1.82 | 7(2) |  | 8.23 ± 0.97 | 9(2) |  |
| Lab staff processing and sample collection/X ray tech | 15.01 ± 3.07 | 15(4) | |  | 6.50 ± 1.49 | 6(2) |  | 8.28 ± 0.94 | 9(2) |  |
| Housekeeping sanitary workers | 14.43 ± 3.49 | 14(6) | |  | 6.26 ± 1.62 | 6(2) |  | 8.62 ± 0.71 | 9(1) |  |
| Ambulance driver/staff/ward boys/guards | 15.16 ± 3.77 | 14(5) | |  | 6.44 ± 1.49 | 6(2) |  | 8.35 ± 1.06 | 9(1) |  |
| ASHA/UHW/USHA | 15.23 ± 3.80 | 14(4) | |  | 6.81 ± 1.94 | 6(3) |  | 8.05 ± 1.09 | 8(2) |  |
| **Employment Status** |  | | |  |  | | |  |  |  |
| Temporary | 15.46 ± 3.84 | 15(5.75) | | 0.89 | 6.78 ± 1.80 | 6(3) | 0.17 | 8.24 ± 1.01 | 9(2) | 0.91 |
| Permanent | 15.36 ± 3.36 | 15(5) | |  | 6.57 ± 1.58 | 6(3) |  | 8.23 ± 1.07 | 9(2) |  |
| **Occupational type** |  | | |  |  | | |  |  |  |
| Medical | 16.08 ± 3.83 | 16(5) | | 0.001 | 7.10 ± 1.83 | 7(2) | 0.001 | 8.06 ± 1.11 | 9(2) | 0.001 |
| Non-medical | 15.03 ± 3.51 | 14(5) | |  | 6.46 ± 1.61 | 6(2) |  | 8.34 ± 0.97 | 9(1) |  |
| **Income levels** |  | | |  |  | | |  |  |  |
| **<20000** | 15.2+/-3.78 | 14(5) | | 0.001 | 6.48+/1.66 | 6(2) | 0.001 | 8.34+/-0.99 | 9(1) | 0.001 |
| **>=20000** | 15.76+/-3.72 | 15(5) | |  | 6.93+/-1.79 | 7(3) |  | 8.10+/1.09 | 9(2) |  |
| **Residing with family** |  | | |  |  | | |  |  |  |
| Yes | 15.25 ± 3.62 | | 14(5) | 0.03 | 6.56 ± 1.63 | 6(2) | 0.001 | 8.28 ± 1.01 | 9(1) | 0.08 |
| No | 15.81 ± 3.75 | | 15(5) |  | 7.04 ± 1.88 | 7(3) |  | 8.14 ± 1.08 | 9(2) |  |
| **Education** |  | | |  |  | | |  |  |  |
| Graduate and above | 15.73 ± 3.78 | | 15(5) | 0.001 | 6.87 ± 1.79 | 6(3) | 0.001 | 8.14 ± 1.06 | 9(2) | 0.001 |
| Below graduate | 14.76 ± 3.34 | | 14(5) |  | 6.34 ± 1.53 | 6(2) |  | 8.44 ± 0.94 | 9(1) |  |
| **Working hours** |  | |  |  |  |  |  |  |  |  |
| <=8 hours | 15.26+/3.69 | | 14(5) | 0.008 | 6.59+/1.70 | 6(3) | 0.01 | 8.23+/1.04 | 9(2) | 0.54 |
| >8 hours | 15.79+/3.60 | | 15(5) |  | 6.96+/1.73 | 7(2) |  | 8.26+/1.05 | 9(1) |  |
