# Supplementary material for: Psychological distress and burnout among healthcare worker during COVID-19 pandemic in India—A cross-sectional study
Source: PLoS One. 2022 Mar 10;17(3):e0264956. doi: 10.1371/journal.pone.0264956 (PMC8912126; doi:10.1371/journal.pone.0264956)
Supplement: Attachment — Submitted filename: Authors response to reviewers comments PONE-D-21-26475.docx [file pone.0264956.s005.docx]

**Author’s response to the Reviewer’s comments**

Please find the comments and their respective responses below

***Reviewer #1 comments and Author’s responses:***

**Comments:** The authors conducted a cross sectional study and explored the psychological distress and burnout rate among the healthcare workers during COVID-19 pandemic. The aim of the study was to determine the burnout levels and factors associated with the risk of psychological distress among healthcare workers (HCW) engaged in the management of COVID-19 in India. This is an interesting representation of data and several significant insights were found which may help in future pandemic management. However, the authors need to address some issues before acceptance of the manuscript.

**Response:** We wish to thank the reviewer for motivating feedback. The authors are grateful to the esteemed reviewer for appreciating the relevance of our work. We have considered all the suggestions given by reviewer# 1 in the revised version of the manuscript.

Our detailed reply to each and every point raised by the Reviewer #1 is given below:

**Query 1:** A table on questionnaire need to be added. Why did the authors specifically choose the GHQ-5 and ICMR-NIOH questionnaires? Why not other questionnaires?

**Response:** Thanks to the reviewer for this important observation. The questionnaire has been attached as a supporting document (S1). The reason for using GHQ-5 and ICMR-NIOH is given below and the same has been incorporated in the revised version of the manuscript.

The GHQ-5, is a shorter screening tool (not a diagnostic tool) which has 5 questions with better discriminators for psychological distress derived from GHQ-12 that involves royalty for usage. GHQ-5 is validated on the Indian population with a sensitivity of 86%, specificity of 95.8% and misclassification rate of 8.3% (Reference). For this study that was done telephonically and needed quick assessment of the psychological state of the respondent with minimum number of questions, GHQ-5 was found to be an effective tool with regard to time and process. It is also available for use in public domain as compared to GHQ-12.

*Reference: C. Shamasunder, T. G. Sriram, S. G. Murali Raj, and V. Shanmugham, “Validity of a short 5-item version of the general health questionnaire (g.h.q).,” Indian J. Psychiatry, vol. 28, no. 3, pp. 217–9, Jul. 1986.*

ICMR-NIOH burnout questionnaire is a shortened and easier version of the Maslach Burnout inventory (MBI) (Reference 1). The questionnaire is a 19 item tool that covers three dimensions of burnout: a) emotional exhaustion (EE, 11 items); (b) depersonalisation (DP, 5 items) and (c) personal accomplishment (PA, 3 items) on a 3-point scale starting from 1,” never,” 2, “sometimes” and 3, “always”. Higher scores on the EE and DP subscales and lower scores on the PA subscale indicate high degrees of experienced burnout. It was developed by the scientists of the ICMR-National Institute of Occupational Health (NIOH) and was under validation for Indian settings during the study. Now it has been validated and published (Reference 2). It was freely available for use whereas the MBI inventory is copyrighted and cannot be reproduced without permission from the developers. Due to the ongoing pandemic and need to make a quick assessment of burnout among healthcare workers, we used the ICMR-NIOH questionnaire.

*Reference 1: C. Maslach, S. E. Jackson, and M. P. Leiter, “Maslach Burnout Inventory: Third edition.,” in Evaluating stress: A book of resources., Lanham, MD, US: Scarecrow Education, 1997, pp. 191–218.*

*Reference 2. S. Balachandar, R., Ketharam, A., & Bharath, “Development and validation of tool for screening occupational mental health and workplace factors influencing it,” PsyArXiv. July 1., pp. 1–17, 2021.*

**Query 2:** The authors did not mention the sampling procedure of the study. It should be mentioned.

**Response:** Thanks for your observation. The detailed sampling procedure and selection of participants has been described in the revised version of the manuscript.

**Query 3:** Several statistical tests have been performed. A short description of tests may help the readers to understand the article even better.

**Response:** Thanks for your observation. The step by step statistical procedures/tests with equation have been added in the statistical analysis section of the revised manuscript.

**Query 4:** There was no city wise distribution of burnout levels and psychological distress rate which need to be done. A comparison with average COVID cases with the burnout and psychological distress may be interesting.

**Response:** The purpose of the study was to gain a quick insight on the psychological state and burnout levels of healthcare workers in India when state wise COVID infection rates were varying. A representative sample in each state was difficult to obtain due to the government restrictions on travel and shorter duration of the study. A minimum sample size of 904 was determined to obtain the distribution at the national level and not at the city/state level. Hence it would be inappropriate to provide the city wise data in the manuscript.

We appreciate the reviewers comment to compare the COVID cases with the burnout levels and psychological distress. However, we could not do this since the data on number of COVID cases was not verified from official records due to the inability of the investigators to physically approach the facility because of ongoing pandemic and lockdown.

**Query 5:** This manuscript lacks visual representation of data. More visual representation may be added which will help the reader to understand the data.

**Response:** We thank the reviewer for the observation. We have displayed the results in graphics (Fig1-6) for better visual representation in the revised manuscript.

Query 6: There were several typos, comma and spacing problems.

**Response:** We appreciate the observation. We have corrected for grammatical errors in the revised version of the paper.

Query 7: Results written in abstract need to be rewritten. It was not clear to the reviewer.

**Response:** We have rewritten the results in the abstract.

**Query 8:** Causes of psychological distress can be shown in pictorial form which may increase the readability of the article.

**Response:** We appreciate the reviewer for this observation. The factors associated with risk of psychological distress has been shown in pictorial form in the revised manuscript Fig3-4.

**Query 9:** Overall writing needs to be improved. Reviewer found difficulty in understanding the result discussion.

**Response:** We appreciate the observation. Overall writing has been improved to bring more clarity in the revised manuscript.

**Query 10**: A specific section for abbreviation or terminology maybe added which will increase the readability.

**Response:** We have added a section on list of acronyms.

***Reviewer’s #2 comments and Authors’ responses:***

**Comment:** Reviewer #2: This paper proposes Psychological Distress and Burnout among Healthcare Worker during COVID-19 Pandemic in India- A cross-sectional study.

**Response:** The authors are grateful to the esteemed reviewer for appreciating the relevance of our work. We have considered all the suggestions given by reviewer# 2 in the revised version.

Our detailed reply to each and every point raised by the Reviewer’s #2 is given below:

**Comment 1:** In this paper the authors took 12 different cities for study purpose but didn’t give any justification of choosing those cities for research purpose.

Response: The study was an ICMR multicentric Task Force project. The sites were chosen based on the response of the site investigators to a call for proposals by the ICMR National Task Force on Operation Research on COVID-19. We selected those implementing agencies who submitted the concept proposal with similar objectives. These agencies were located in 10 states that broadly represented different regions of the country. Due to the lockdown rules and travel regulations in different cities, from each state one city was selected where the implementing agency was located except in Kerala and Odisha where two cities were selected as per the convenience of site investigators.

**Comment 2:** The authors failed to mention what time of a day they have conducted the interview which may have a major impact while researching on psychological impact of healthcare workers for this study.

**Response:** Thanks for your observation. As mentioned in the revised manuscript the investigators fixed an appointment with the healthcare workers as per their convenience so as to ensure that their duty time or leisure time was not disturbed.

**Comment 3:** The authors should include a functional diagram in the paper to explain the flow of the work as well as mathematical modelling to explain the multiple logistic regression process that has been used for adjusting the risk factors.

Response: We have added the functional diagram (S2) and have described the mathematical modelling procedures in the statistical analysis section of the revised manuscript.

**Comment 4:** All the figures are too blur to understand and therefore should be redraw.

**Response:** We appreciate the observation of the reviewer and all the figures have been redrawn Fig1-6.

**Comment 5:** There are grammatical errors in the paper which reduce the readability of the article. These errors should be corrected.

**Response:** We appreciate the observation. We have corrected for grammatical errors in the revised version of the paper.
