# Supplementary material for: Psychological distress and burnout among healthcare worker during COVID-19 pandemic in India—A cross-sectional study
Source: PLoS One. 2022 Mar 10;17(3):e0264956. doi: 10.1371/journal.pone.0264956 (PMC8912126; doi:10.1371/journal.pone.0264956)
Supplement: Attachment — Submitted filename: Authors response to reviewers comments PONE-D-21-26475 R2.docx [file pone.0264956.s006.docx]

**Author’s response to the Reviewer’s comments**

Please find the comments and their respective responses below

**Comments to the Author**

1. If the authors have adequately addressed your comments raised in a previous round of review and you feel that this manuscript is now acceptable for publication, you may indicate that here to bypass the “Comments to the Author” section, enter your conflict of interest statement in the “Confidential to Editor” section, and submit your "Accept" recommendation.

Reviewer #1: All comments have been addressed

Reviewer #2: All comments have been addressed

Response: Thanks for the reviewer’s appreciation

2. Is the manuscript technically sound, and do the data support the conclusions?

The manuscript must describe a technically sound piece of scientific research with data that supports the conclusions. Experiments must have been conducted rigorously, with appropriate controls, replication, and sample sizes. The conclusions must be drawn appropriately based on the data presented.

Reviewer #1: Yes

Reviewer #2: Yes

Response: Thanks for the reviewer’s appreciation

3. Has the statistical analysis been performed appropriately and rigorously?

Reviewer #1: Yes

Reviewer #2: Yes

Response: Thanks for the reviewer’s appreciation

4. Have the authors made all data underlying the findings in their manuscript fully available?

The [PLOS Data policy](http://www.plosone.org/static/policies.action#sharing XztRf1NYBDQBPVRmB2gANlg8) requires authors to make all data underlying the findings described in their manuscript fully available without restriction, with rare exception (please refer to the Data Availability Statement in the manuscript PDF file). The data should be provided as part of the manuscript or its supporting information, or deposited to a public repository. For example, in addition to summary statistics, the data points behind means, medians and variance measures should be available. If there are restrictions on publicly sharing data—e.g. participant privacy or use of data from a third party—those must be specified.

Reviewer #1: No

Response: Thanks for the observation. We have now uploaded the data as per the PLOS DATA POLICY

Reviewer #2: Yes

Response: Thanks for the valuable comments

5. Is the manuscript presented in an intelligible fashion and written in standard English?

PLOS ONE does not copyedit accepted manuscripts, so the language in submitted articles must be clear, correct, and unambiguous. Any typographical or grammatical errors should be corrected at revision, so please note any specific errors here.

Reviewer #1: Yes

Reviewer #2: Yes

Response: Thanks for the reviewer’s appreciation

6. Review Comments to the Author

Please use the space provided to explain your answers to the questions above. You may also include additional comments for the author, including concerns about dual publication, research ethics, or publication ethics. (Please upload your review as an attachment if it exceeds 20,000 characters)

Reviewer #1: I am satisfied with how the authors addressed my comments. With some minor revisions, I accept the manuscript. Make sure that every figure is clear and well-illustrated. Captions should be self-explanatory. Particularly, Figures 3, 4, and 5 are blurry and difficult to read.

Response: Thanks for the accepting the manuscript. The authors appreciate the reviewers for their valuable inputs. We have now uploaded then revised figures.

Reviewer #2: (No Response)

Response: Thanks to the reviewer.
